# Direct Rub Inoculation of Fungal Flora Changes Fatty Acid Composition and Volatile Flavors in Dry-Aged Beef: A Preliminary Study

**DOI:** 10.3390/ani12111391

**Published:** 2022-05-28

**Authors:** Nana Mikami, Takahito Toyotome, Masahiro Takaya, Kenichi Tamura

**Affiliations:** 1 Department of Life and Food Sciences, Obihiro University of Agriculture and Veterinary Medicine, Obihiro 080-8555, Hokkaido, Japan; 2 Department of Veterinary Medicine, Obihiro University of Agriculture and Veterinary Medicine, Obihiro 080-8555, Hokkaido, Japan; tome@obihiro.ac.jp; 3 Diagnostic Center for Animal Health and Food Safety, Obihiro University of Agriculture and Veterinary Medicine, Obihiro 080-8555, Hokkaido, Japan; 4 Medical Mycology Research Center, Chiba University, Chiba 260-8673, Japan; 5 The Tokachi Foundation, Obihiro 080-2462, Hokkaido, Japan; takaya@food-tokachi.jp; 6 Kitaichi Meat Co., Ltd., Sapporo 007-0826, Hokkaido, Japan; k-tamura@kitaichimeat.com

**Keywords:** direct rub inoculation, dry-aged beef, *Mucoraceae*, oleic acid, volatile aromatic compounds, nutty flavor

## Abstract

**Simple Summary:**

Dry-aged beef naturally develops a crust of desirable fungi and bacteria over the aging process. However, this natural growth process is expensive and time-consuming. The process of direct fungal adhesion may accelerate aging and improve the flavor and fatty acid profile of dry-aged beef. We compared the fungal growth and meat quality of dry-aged beef inoculated by natural fungal adherence and dry-aged beef inoculated by direct application of fungi (direct adhesion) to assess the differences in appearance, volatile flavors, and fatty acid composition. After 26 days of aging, dry-aged beef that received direct fungal application exhibited increased surface mold, volatile compound intensity, and oleic acid composition compared with the dry-aged beef that did not receive direct inoculation. Thus, we report an improved method of manufacturing dry-aged beef with high quality that has the potential to enhance the “melt-in-the-mouth” feeling and flavors.

**Abstract:**

Here, we established a method to produce dry-aged beef (DAB) by rub inoculation with fungal flora on the prepared DAB surface. Portions of Holstein steers’ rumps were prepared by direct rub inoculation of fungal flora or without treatment (conventional DAB) and dry-aged for 26 days in an aging room at 2.9 °C and 90% relative humidity. We compared the fungal covering and meat quality, including fatty acid composition and volatile aromatic compounds, of fungal-inoculated DAB with those of the conventional DAB. The fungal-inoculated DAB was almost entirely covered with white mold, in contrast to the conventional DAB. Moreover, the proportion of oleic acid and the concentration of nine volatile compounds significantly increased in the raw meat of fungal-inoculated DAB compared with those in the conventional DAB (*p* < 0.05). These results suggested that direct rub inoculation of fungal flora from prepared DAB may accelerate DAB production and efficiently enhance the “melt-in-the-mouth” feeling and flavors of DAB.

## 1. Introduction

In dry-aging, a traditional method used to preserve meat, the meat surface is exposed to air flow under regulated temperature and relative humidity conditions. Dry-aged beef (DAB) develops a dried surface on the portions of meat, a “crust”, which is comprised of a variety of microorganisms, including fungi and bacteria [1]. Although reports are still limited, it has been focusing on an interaction between microflora on the crust and the dry-aging process [2,3,4]. Previous studies have shown that the fungi belonging to the *Mucoraceae* family are notably prominent in the crust of DAB [5,6]. The presence of *Mucoraceae* members is considered to enhance the flavor compounds, amino acids, and fatty acids in DAB owing to the production of proteolytic and lipolytic enzymes [6]. Therefore, efficient and stable growth of these molds on DAB is desired.

DAB is conventionally manufactured using fungi that naturally cover the meat in an aging room [2,7]. However, this approach does not enable the selection of species or the amount of fungi, and this results in unstable qualities of DAB. Moreover, conventional methods of causing fungi to passively adhere to the meat and allowing them to naturally grow is a time-consuming and expensive process, as sufficient growth requires a long aging period. In order to manufacture DAB efficiently, several inoculation methods have been tried recently. Hanagasaki and Asato reported that a mold strain cultured on potato dextrose agar plates was directly brought into contact with pieces of beef rump meat [5]. Oh et al. [6] also reported that the fungal suspension was spread evenly onto the surface of beef sirloin by spraying. Compared to the conventional DAB production method, these direct inoculation methods are expected to enable the growth of the targeted microorganism with a high probability. However, to the best of our knowledge, there have been no studies comparing the effects of the conventional and the direct inoculation strategies on fungal growth and the quality of DAB. We hypothesized that active fungi inoculation onto the meat could facilitate the production of DABs over conventional methods of naturally adhering fungi. Therefore, as a preliminary study, we aimed to compare fungal growth and the meat quality between DAB inoculated via natural fungal adherence (conventional) and that inoculated via active transfer of fungi from the prepared DAB crust (direct rub inoculation) to assess the differences in appearance, fatty acid composition, and volatile aromatic compounds.

## 2. Materials and Methods

### 2.1. Production of Dry-Aged Beef

Boneless rump (*Musculus gluteus medius*, *Musculus gluteus profundus*, and *Musculus biceps fermoris* were joined together) obtained from one Holstein steer (19 months old) was used for dry-aging in this study. The rump was cut into half parallel to the long side direction so that the weights of the two cut portions were as equal as possible (approximately 4.4 kg/portion) (we did not consider the composition of the anatomical muscle types contained in the two portions in this study). Only at the beginning of aging was direct rub inoculation performed evenly on the surface of one portion of the rump. We used a crust on a small piece of DAB meat (approximately 5 × 5 × 10 cm) for the rub inoculation, which was produced in the same aging room (Goshima Thermal Engineering Co., Ltd., Hokkaido, Japan) as reported in our previous study [8]. The crust was covered with fungal flora, including *Mucor flavus*, *Helicostylum pulchrum*, and *Penicillium* series *Camembertiorum*. The other portion was untreated (conventional DAB). Both portions were dry-aged for 26 days by placing them on a stainless rack in the same aging room set up at Kitaichi Meat Co., Ltd. (Hokkaido, Japan). The aging room was routinely used for DAB production; other DABs not used in this study were also aged in the same room. We allowed the fungal flora in the room air to contact both portions during aging. The aging conditions were as follows: average temperature, 2.9 °C; relative humidity, 90%; and airflow, 1.8–2.5 m/s. After aging, the beef portions were transported under refrigeration to the laboratory. The crusts were trimmed off from both meat portions. Steaks of 1-inch (2.54 cm) thickness were sampled from the portions and used to measure cooking loss and shear force. The remaining meat was cut into 2–3 cm sized pieces, minced, and then sampled in plastic tubes. The samples were stored at −30 °C until analyses.

Aging and trimming losses were calculated as a percentage of the loss to the weight of the meat portion before the start of aging by subtracting the weights before and after aging and trimming, respectively. Briefly, aging loss (%) = (weight before aging − weight after aging)/weight before aging ×100 and trimming loss (%) = (weight before trimming − weight after trimming)/weight before aging × 100.

### 2.2. Observation of the Appearance of Dry-Aged Beef during the Aging Process

At 7, 13, and 20 days after the start of dry-aging, the growth of fungi on the cut surface was visually observed with (+) or without (−) direct fungal-rub inoculation. On the 26 th day, the images of the entire portion were compared between the two groups.

### 2.3. Proximate Composition

Proximate composition, including moisture, crude protein, crude fat, and ash content, of DAB with and without fungal flora-rub inoculation was determined according to the methods of the Association of Official Analytical Chemists (AOAC) (2002). Moisture content was measured according to AOAC method 934.01. Crude protein and fat content were determined using the Kjeldahl (AOAC method 928.08) and Soxhlet (AOAC method 991.36) methods, respectively. The ash content was determined using a muffle furnace at 550 °C (AOAC method 923.03).

### 2.4. Water-Holding Capacity and Shear Force Measurements

Expressible drip loss was measured as described by Hamm [9], with slight modifications. Briefly, 1 g of minced DAB with and without fungal-rub inoculation was placed between two 100-mesh nylon filters sandwiched by four sheets of previously weighed filter paper (No. 526, Toyo Roshi Kaisha, Ltd., Tokyo, Japan). The assembly of the force gauge (Marubishi Kagaku Kikai Factory, Tokyo, Japan) was subjected to 30 kg of pressure for 1 min. After pressing, the filter papers were reweighed. The expressible drip loss was calculated as the percentage of weight gain of the filter papers.

Cooking loss was measured using the method of Honikel [10], with a slight modification. One-inch steak samples were weighed and placed inside a plastic bag and incubated for 60 min at 70 °C in a water bath (SP-12R, Taitec Corporation, Saitama, Japan). The samples were cooled, patted dry with paper towels, and then reweighed. The difference between the weights before and after incubation was calculated and expressed as a ratio relative to the weight before incubation.

After determining cooking loss, the incubated steak was wrapped in a polyvinylidene chloride film and stored overnight at 4 °C. Thereafter, 11 cores were taken from the steak using a 1.27-cm-diameter coring device situated to collect samples parallel to the longitudinal axis of the muscle fiber. The cores were then sheared using a Warner–Bratzler meat shear machine (GR Manufacturing Company, Manhattan, KS, USA) equipped with V style Warner–Bratzler blade (operating at a constant speed (225 mm/min)). The Warner–Bratzler shear force (WBSF) of the steak was calculated as the average maximum force (kg) required to shear through each sample.

### 2.5. Fatty Acid Analysis

The total lipids were extracted from 0.5 g of minced raw meat from the DAB with or without fungal flora-rub inoculation, using the Bligh and Dyer [11] method. Methylation of the total lipids was performed using the method of Prevot and Mordret [12]. Briefly, the total lipids were dissolved in 1 mL of *n*-hexane in a test tube, and 0.2 mL of 2 N MeOH–NaOH was added and mixed before incubation at 50 °C for 30 s. Thereafter, 0.2 mL of 2 N MeOH-HCl was added. After centrifuging the solution centrifuged at 3000 rpm for 5 min, the upper (*n*-hexane) layer was collected as fatty acid methyl esters (FAMEs). The FAMEs were analyzed by gas chromatography–flame ionization detector (SHIMADZU GC-2014) equipped with a Zebron ZB-FAME capillary column (30 m × 0.25 mm I.D., 0.20 μm film thickness; Phenomenex Inc., Torrance, CA, USA) using nitrogen as the carrier gas (1.3 mL/min). The injection temperature was 250 °C, and the detector was set at 260 °C. The column temperatures were increased from 140 °C to 240 °C at a rate of 4 °C/min and held at 240 °C for 15 min. The FAMEs were identified by comparing them to the Supelco 37 Component FAME standards (Merck KGaA, Darmstadt, Germany).

### 2.6. Volatile Aromatic Compound Analysis

Approximately 1 g of minced raw meat from DAB with or without the fungal-rub inoculation was placed into 15 mL glass vials with a PTFE/silicone septum and stored at −80 °C until analysis. The volatile compounds were prepared and then analyzed using a gas chromatograph-mass spectrometer (SHIMADZU GC-MS-QP2010) according to a previously described method [8]. The obtained mass spectra were deconvoluted using AMDIS GC/MS Analysis (version 2.73) (The National Institute of Standards and Technology (NIST), Gaithersburg, MD, USA) and matched to those in the Massbank of North America GC-MS Spectra. Additionally, the mass spectra were compared with those in the commercial GC-MS libraries, such as NIST05 and NIST05s, using Shimadzu GC-MS solution software.

### 2.7. Statistical Analysis

All continuous data are expressed as mean ± standard deviation. Means were compared with Student *t*-tests using JMP 13 (SAS Institute Inc., Cary, NC, USA) to identify differences between DAB with (*n* = 3) and without (*n* = 3) direct fungal flora-rub inoculation. Results with *p* < 0.05 were considered statistically significant.

## 3. Results and Discussion

### 3.1. Appearance and Yield of DAB

**Figure 1:**
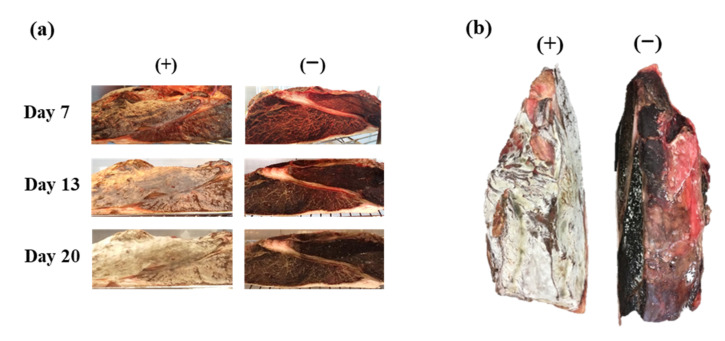
The appearance of dry-aged beef, with (+) and without (−) direct fungal-rub inoculation. (**a**) Cross-section of beef dry-aged for 7, 13, and 20 days. (**b**) Whole portion of beef dry-aged for 26 days.

After dry-aging, the appearance of conventionally prepared and fungal rub-inoculated DAB was visually compared (Figure 1). Fluffy molds were observed on day 7, and the white fluffy area increased by day 20 on the surface of the portion rubbed with fungal flora compared with that in the conventional DAB (Figure 1a, left). As shown in the top view of the portion (Figure 1b, left), almost the entire surface, except the fat, was covered with molds. On the contrary, small mold speckles appeared on day 13 after appearing on the surface of conventional DAB (Figure 1a,b, right). These findings indicated that active rub inoculation of fungal flora on the beef rump portion significantly increased the fungal proportion and promoted fungal colonization on the surface of meat compared with the conventional DAB procedure.

Aging loss of DAB after 26 days with (+) and without (−) fungal-rub inoculation was 17.2% and 15.4%, respectively. These levels were similar to a previous report on DABs inoculated for 4 weeks with and without mold (approximately 15% to 18%) [5]. The trim loss of (+) and (−) DABs was 28.5% and 23.5%, respectively. These results suggested that 5% of more parts have to be trimmed in DAB (+) than in DAB (−). The yield of the final edible meat of DABs was as follows: 59.2% in DAB (+) and 64.7% in DAB (−), respectively.

### 3.2. Proximate Composition

The moisture, crude protein, and crude fat content were determined in DAB (+) and (−). Fungal-rub inoculation significantly decreased the moisture content in DAB (+) (58.9% ± 1.5%) compared with that in DAB (−) (63.9% ± 0.5%) (Table 1). Although Hanagasaki and Asato [5] reported that the mold strain possibly prevented water evaporation from the meat of DAB, we did not observe any such effect in the present study. As fungi require moisture for their growth [13], the inoculated fungi on the crust of DAB (+) may also have used moisture. The crude fat content was higher in DAB (+) (20.1% ± 1.0%) than in DAB (−) (13.4% ± 0.1%) (Table 1). In contrast, there was no significant difference in crude protein content between the (+) and (−) DABs (Table 1). We preliminarily confirmed that there was no difference in the crude fat content of the two portions of Holstein steer’s fresh rumps cut into half in the same manner as in this study (data not shown). However, we could not evaluate the content of the portions used in this study at the beginning of the aging. A previous study showed that the crude fat contents were different between different muscle types [14]. In the present study, we did not consider the composition of the anatomical muscle types contained in the DAB (+) and (−) portions. Therefore, other than direct rub inoculation, it may be possible that differences in the original proximate composition of the two portions before the dry-aging contribute to these results.

### 3.3. Water-Holding Capacity and Shear Force

Water-holding capacity is one of the most important factors affecting the juiciness and tenderness of meat [15]. We evaluated the water-holding capacities of DABs with and without rub inoculation by measuring expressible drip and cooking loss. There was no significant difference in expressible drip loss between the DABs (Table 1). On the contrary, the cooking loss in the DAB (+) was more than that in DAB (−) (Table 1). It has been reported that cooking loss is the same in normal-aged beef (dry-aged without mold; 28.2%) and mold-aged (dry-aged with mold; 28.3%) [5]. Our result is not consistent with the findings of the previous study [5]; our result suggested that direct rub inoculation increases cooking loss in DAB.

The WBSF is the main tool used to evaluate beef tenderness [16], and its values correlate well with the tenderness sensory rating made by consumers [17]. To evaluate whether fungal-rub inoculation tenderizes the DABs, we measured the WBSF of DAB with and without rub inoculation. It is known that *Thamnidium*, which is the mold closest to *Helicostylum* sp. observed in our study, releases proteases that tenderize aging meat (Standards and Guidelines, PrimeSafe). Therefore, we expected that the fungal growth after rub inoculation would reduce the WBSF of the DAB (+). However, in this study, the WBSF of DAB (+) was significantly higher than that of DAB (−) (2.64 vs. 1.68 kg/cm^2^, Table 1). In beef, the cooking loss has been shown to be positively correlated with the WBSF [18]. As shown in Table 1, an increase in the cooking loss, that is, reduced water-holding capacity, in DAB (+) may have contributed to the increase in the WBSF and made it difficult to detect the tenderizing effect of mold on the DAB. On the contrary, as most consumers recognize that meat with a WBSF of 3.36 kg or less is tender or very tender [17], both DABs produced in our study may be considered tender.

### 3.4. Fatty Acid Composition

We evaluated the effect of direct fungal flora-rub inoculation on the fatty acid composition of raw DABs. Among the 18 fatty acids detected via GC, there was a significant difference in the composition of 13 fatty acids between DAB (+) and DAB (−) (Table 2). The three major fatty acids in both DABs were 16:0 (palmitic acid), 18:0 (stearic acid), and 18:1n-9 (oleic acid), which is consistent with the findings of a previous study of fungal-grown DABs [2]. Oleic acid, which is the most abundant monounsaturated fatty acid (MUFA) in the DABs, has a lower melting point than the saturated fatty acids (SFA) [19], which relates to its “melt-in-the-mouth” characteristic. Additionally, oleic acid in meat has been shown to correlate with the flavor of beef [20]. Interestingly, in our study, the oleic acid content was significantly higher in DAB (+) (47.21 ± 0.66%) than in DAB (−) (45.14 ± 0.24%) (Table 2, *p* < 0.01). This result suggested that the direct rub inoculation of fungal flora may increase the flavor of DAB. On the other hand, Alabiso et al. [21] previously showed that the muscle type with high fat content has a high proportion of oleic acid. Because the DAB (+) had a higher crude fat content than the DAB (−), these factors may also affect the high composition of oleic acid in DAB (+), other than direct rub inoculation.

Previous reports showed that increased fat hardness was associated with increased 18:1n-9 and decreased 18:2n-6 percentage [22]. In our results, the percentage of 18:1n-9 in DAB (+) was higher than that in DAB (−), while that of 18:2n-6 in DAB (+) was lower than that in DAB (−) (Table 2). These results may contribute to the higher hardness by WBSF in DAB (+) than in DAB (−) (Table 1).

The proportion of most SFAs (12:0, 14:0, 15:0, 16:0, 17:0, and 18:0) and the total SFA were significantly lower in DAB (+) than in DAB (−) (Table 2). Conversely, the levels of some MUFAs (14:1n-5, 16:1n-7, 17:1n-7, and 18:1n-9) and the total MUFA were higher in DAB (+) than in DAB (−) (Table 2). Moreover, the 16:1/16:0 and 18:1/18:0 ratios were significantly higher in DAB (+) than in DAB (−) (Table 2). The content (mg/g meat) of each of the 18 fatty acids was higher in DAB (+) than in DAB (−) (data not shown). According to a previous study, D^9^-desaturase, one of the well-known desaturases, introduces a double bond at the D^9^-position of SFAs such as palmitic acid (16:0) and stearic acid (18:0) to form MUFAs such as palmitoleic acid (16:1n-7) and oleic acid (18:1n-9), respectively [23]. This enzyme is widely found in different organisms, including animals and fungi [24]. It is still unclear how this enzyme is related to the findings of the present study, although the results suggested that the presence of fungi due to direct rub inoculation may be involved in the desaturation of fatty acids in the DAB. Additionally, as previously reported, a low proportion of SFA and a high proportion of MUFA has also been shown to be associated with the high fat content in muscle [21]. From these findings, it is possible that not only the direct rub inoculation but also the higher crude fat content in the portion of the DAB (+) may be related to the proportion of fatty acids DAB (+) in this study.

### 3.5. Volatile Aromatic Compound Analysis

**Figure 2:**
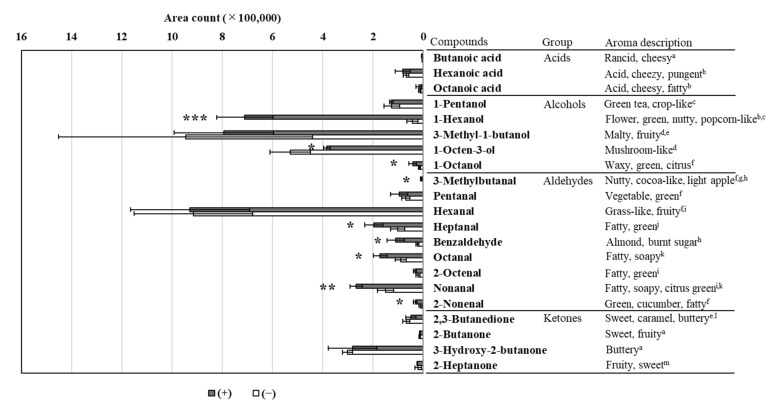
Area count of volatile aromatic compounds produced by raw meat of dry-aged beef, with (+) or without (−) direct fungal-rub inoculation, after 26 days. Values are expressed as mean ± standard deviation (n = 3). *** *p* < 0.001; ** *p* < 0.01; * *p* < 0.05. The following literature was used as reference for aroma descriptions: ^a^ [37]; ^b^ [25]; ^c^ [26]; ^d^ [38]; ^e^ [39]; ^f^ [29]; ^g^ [27]; ^h^ [28]; ^i^ [30]; ^j^ [31]; ^k^ [32]; ^l^ [40]; and ^m^ [41].

By GC-MS, we identified 21 volatile compounds in the raw meat of DAB (+) and DAB (−) after 26 days of aging (Figure 2). According to the four groups of compounds, the peak counts of acids and ketones were not different between DAB (+) and DAB (−). On the contrary, among alcohols, the peak count of 1-hexanol was substantially increased in DAB (+) (7.1 × 10^5^ counts) compared with that in DAB (−) (4.3 × 10^4^ counts) (16.4 times higher, *p* < 0.001). Studies have characterized 1-hexanol as a volatile compound described as “flower,” “green,” “nutty,” and “popcorn-like” [25,26]. Therefore, our results suggested that direct fungal-rub inoculation may impart these aromas to DAB. Moreover, among aldehydes, the peak counts of six compounds (3-methylbutanal, heptanal, benzaldehyde, octanal, nonanal, and 2-nonenal) were significantly higher in DAB (+) than in DAB (−). Notably, 3-methylbutanal and benzaldehyde have been reported to have “nutty” [27], “almond,” and “burned sugar”-like odors [28]. Nutty and brown roasted-like odors are used as a common and representative aroma description that characterizes DAB [7]. The other four compounds (heptanal, octanal, nonanal, and 2-nonenal) have been commonly described as “fatty” and “green” in previous reports [29,30,31,32]. Utama et al. [33] showed that dry-aging increased the intensity of multiple aldehydes (3-methylbutanal, heptanal, benzaldehyde, octanal, and nonanal). These findings support that the increase in the levels of some aldehydes in DAB upon direct fungal-rub inoculation may enhance the characteristics of DAB. Furthermore, these compounds have been reported to be produced in meat products by microorganisms, particularly fungi [34,35,36]. Therefore, our results suggested that direct fungal-rub inoculation may enhance aromatic compound production via fungal growth promotion on DAB. In our results, the crude fat content was significantly higher in DAB (+) than in DAB (−). Previous reports described that the chemical breakdown of fat resulted in a more nutty and beefy flavor [7]. To clarify the effect of direct rub inoculation in more detail, further research is required under conditions where the fat content in DAB is controlled.

## 4. Conclusions

We preliminarily evaluated the effect of direct inoculation of fungal flora by rubbing prepared DAB crust on the fungal growth, meat quality, fatty acid composition, and volatile aromatic compounds of DAB. Fungal flora-rub inoculation significantly altered the appearance of DAB, which was almost entirely covered with white mold after the 26-day aging period. The composition of oleic acid, which may be associated with flavor and “melt-in-the-mouth” characteristic of meat, was significantly higher in DAB subjected to fungal inoculation than in the conventional DAB. Furthermore, the production of nine volatile aromatic compounds was enhanced in the fungal-inoculated DAB. Notably, 1-hexanol, which was previously described as “flower”, “green”, “nutty”, and “popcorn-like” flavor, was considerably increased. These results suggested that direct fungal inoculation may effectively accelerate the production of DAB and efficiently enhance the volatile aroma compound in DAB. In this study, there are some limitations because the tested portions may not contain three types of muscle in the rump equally. Further research is needed to clarify the action of each fungus rub-inoculated from fungal flora on meat characteristics of DAB.

## Figures and Tables

**Table 1:** Proximate composition, water-holding capacity, and tenderness of 26-day dry-aged beef with (+) and without (−) direct fungal adhesion.

Characteristics	+	−
Moisture (%)	58.9 ± 1.5 **	63.9 ± 0.5
Crude protein (%)	20.8 ± 0.2	21.5 ± 0.7
Crude fat (%)	20.1 ± 1.0 **	13.4 ± 0.1
Ash (%)	1.03 ± 0.02 **	1.14 ± 0.04
Expressible drip loss (%)	31.2 ± 2.3	27.3 ± 0.9
Cooking loss (%)	26.0 ± 0.3 **	16.6 ± 2.2
WBSF (kg/cm^2^)	2.64 ± 0.32 *	1.68 ± 0.17

Values are expressed as mean ± standard deviation (n = 3). ** *p* < 0.01 vs. without (−); * *p* < 0.05 vs. without (−). WBSF, Warner–Bratzler shear force.

**Table 2:** Fatty acid composition of 26-day dry-aged beef with (+) and without (−) direct fungal adhesion.

FAME (%)	With (+)	Without (−)
8:0	0.02 ± 0.00	0.03 ± 0.00
12:0	0.04 ± 0.00 *	0.04 ± 0.00
13:0	0.01 ± 0.00	0.01 ± 0.00
14:0	2.44 ± 0.01 *	2.63 ± 0.08
14:1n-5	0.70 ± 0.02 *	0.64 ± 0.02
15:0	0.44 ± 0.01 *	0.47 ± 0.01
15:1n-5	0.11 ± 0.00	0.12 ± 0.00
16:0	24.25 ± 0.09 **	25.20 ± 0.24
16:1n-7	4.51 ± 0.04 **	4.21 ± 0.08
17:0	1.32 ± 0.02 **	1.40 ± 0.01
17:1n-7	1.25 ± 0.02 **	1.16 ± 0.02
18:0	11.09 ± 0.40 *	11.97± 0.15
18:1n-9(*cis*)	47.21 ± 0.66 **	45.14 ± 0.24
18:2n-9	0.44 ± 0.00 **	0.47 ± 0.00
18:2n-6	2.65 ± 0.09 **	2.99 ± 0.06
18:3n-3	0.11 ± 0.00	0.11± 0.00
20:0	0.35 ± 0.00	0.34 ± 0.00
20:4n-6	0.29 ± 0.03 **	0.40 ± 0.03
SFA	39.96 ± 0.49 **	42.09 ± 0.34
MUFA	53.79 ± 0.64 **	51.27 ± 0.16
PUFA	3.49 ± 0.12 **	3.98 ± 0.09
16:1/16:0 ratio	0.19 ± 0.00 ***	0.17 ± 0.00
18:1/18:0 ratio	4.26 ± 0.21 *	3.77 ± 0.05

Values are expressed as mean ± standard deviation (n = 3). *** *p* < 0.001 vs. without (−); ** *p* < 0.01 vs. without (−); * *p* < 0.05 vs. without (−). SFA, saturated fatty acids; MUFA, monounsaturated fatty acids; PUFA, polyunsaturated fatty acids.

## Data Availability

The data presented in this study are available on request from the corresponding author.

## Further Reading

Standards and Guidelines. Ageing of Beef. Available online: https://www.primesafe.vic.gov.au/standards-and-guidelines/primenotes/ageing-of-beef/ (accessed on 14 December 2021).

## References

[1] Ryu S., Park M.R., Maburutse B.E., Lee W.J., Park D.-J., Cho S., Hwang I., Oh S., Kim Y. (2018). Diversity and characteristics of the meat microbiological community on dry aged beef. J. Microbiol. Biotechnol..

[2] Lee H.J., Yoon J.W., Kim M., Oh H., Yoon Y., Jo C. (2019). Changes in microbial composition on the crust by different air flow velocities and their effect on sensory properties of dry-aged beef. Meat Sci..

[3] Endo A., Koizumi R., Nakazawa Y., Shiwa Y., Maeno S., Kido Y., Irisawa T., Muramatsu Y., Tada K., Yamazaki M. (2021). Characterization of the microbiota and chemical properties of pork loins during dry aging. Microbiologyopen.

[4] Ryu S., Shin M., Cho S., Hwang I., Kim Y., Oh S. (2020). Molecular Characterization of microbial and fungal communities on dryaged beef of Hanwoo using metagenomic analysis. Foods.

[5] Hanagasaki T., Asato N. (2018). Changes in free amino acid content and hardness of beef while dry-aging with *Mucor flavus*: Changes in the quality of beef while dry-aging with *Mucor flavus*. J. Anim. Sci. Technol..

[6] Oh H., Lee H.J., Lee J., Jo C., Yoon Y. (2019). Identification of microorganisms associated with the quality improvement of dry-aged beef through microbiome analysis and DNA sequencing, and evaluation of their effects on beef quality. J. Food Sci..

[7] Dashdorj D., Tripathi V.K., Cho S., Kim Y., Hwang I. (2016). Erratum to: Dry aging of beef; Review. J. Anim. Sci. Technol..

[8] Mikami N., Toyotome T., Yamashiro Y., Sugo K., Yoshitomi K., Takaya M., Han K.-H., Fukushima M., Shimada K. (2021). Dry-aged beef manufactured in Japan: Microbiota identification and their effects on product characteristics. Food Res. Int..

[9] Hamm R., Bechtel P.J. (1986). Functional properties of the myofibrillar system and their measurements. Muscle as Food.

[10] Honikel K.O. (1998). Reference methods for the assessment of physical characteristics of meat. Meat Sci..

[11] Bligh E.G., Dyer W.J. (1959). A rapid method of total lipid extraction and purification. Can. J. Biochem. Physiol..

[12] Prevot A.F., Mordret F.X. (1976). Use of glass capillary columns for the analysis of fatty substances by gas chromatography. Rev. Fr. Corps Gras..

[13] Van Long N.N., Rigalma K., Coroller L., Dadure R., Debaets S., Mounier J., Vasseur V. (2017). Modelling the effect of water activity reduction by sodium chloride or glycerol on conidial germination and radial growth of filamentous fungi encountered in dairy foods. Food Microbiol..

[14] Maniaci G., Alabiso M., Francesca N., Giosue C., Di Grigoli A., Corona O., Cardamone C., Graci G., Portolano B., Bonanno A. (2020). Bresaola made from Cinisara cattle: Effect of muscle type and animal category on physicochemical and sensory traits. CyTA J. Food.

[15] Irie M., Izumo A., Mohri S. (1996). Rapid method for determining water-holding capacity in meat using video image analysis and simple formulae. Meat Sci..

[16] Silva D.R.G., de Moura A.P.R., Ramos A.L.S., Ramos E.M. (2017). Comparison of Warner-Bratzler shear force values between round and square cross-section cores for assessment of beef *Longissimus* tenderness. Meat Sci..

[17] Destefanis G., Brugiapaglia A., Barge M.T., Dal Molin E. (2008). Relationship between beef consumer tenderness perception and Warner-Bratzler shear force. Meat Sci..

[18] Silva J.A., Patarata L., Martins C. (1999). Influence of ultimate pH on bovine meat tenderness during ageing. Meat Sci..

[19] Nakahashi Y., Maruyama S., Seki S., Hidaka S., Kuchida K. (2008). Relationships between monounsaturated fatty acids of marbling flecks and image analysis traits in longissimus muscle for Japanese Black steers. J. Anim. Sci..

[20] Westerling D.B., Hedrick H.B. (1979). Fatty acid composition of bovine lipids as influenced by diet, sex and anatomical location and relationship to sensory characteristics. J. Anim. Sci..

[21] Alabiso M., Maniaci G., Giosuè C., Gaglioa G., Francesca N., Di Grigoli A., Portolano B., Bonanno A. (2020). Effect of muscle type and animal category on fatty acid composition of bresaola made from meat of Cinisara cattle: Preliminary investigation. CyTA J. Food.

[22] Maw S.J., Fowler V.R., Hamilton M., Petchey A.M. (2003). Physical characteristics of pig fat and their relation to fatty acid composition. Meat Sci..

[23] Stukey J.E., McDonough V.M., Martin C.E. (1989). Isolation and characterization of OLE1, a gene affecting fatty acid desaturation from *Saccharomyces cerevisiae*. J. Biol. Chem..

[24] Shanklin J., Somerville C. (1991). Stearoyl-acyl-carrier-protein desaturase from higher plants is structurally unrelated to the animal and fungal homologs. Proc. Natl Acad. Sci. USA.

[25] Mu S., Liu L., Liu H., Shen Q., Luo J. (2021). Characterization of the relationship between olfactory perception and the release of aroma compounds before and after simulated oral processing. J. Dairy Sci..

[26] Migita K., Iiduka T., Tsukamoto K., Sugiura S., Tanaka G., Sakamaki G., Yamamoto Y., Takeshige Y., Miyazawa T., Kojima A. (2017). Retort beef aroma that gives preferable properties to canned beef products and its aroma components. Anim. Sci. J..

[27] Luo J., Jiang C., Zhao L., Zhang M., Wang F., Sun E., Ren F. (2018). Keto acid decarboxylase and keto acid dehydrogenase activity detected during the biosynthesis of flavor compound 3-methylbutanal by the nondairy adjunct culture *Lactococcus lactis* ssp. lactis F9. J. Dairy Sci..

[28] Yu H., Xie T., Xie J., Ai L., Tian H. (2019). Characterization of key aroma compounds in Chinese rice wine using gas chromatography-mass spectrometry and gas chromatography-olfactometry. Food Chem..

[29] Feng T., Shui M., Song S., Zhuang H., Sun M., Yao L. (2019). Characterization of the key aroma compounds in three truffle varieties from China by flavoromics approach. Molecules.

[30] Bi S., Xu X., Luo D., Lao F., Pang X., Shen Q., Hu X., Wu J. (2020). Characterization of key aroma compounds in raw and roasted peas (*Pisum sativum* L.) by application of instrumental and sensory techniques. J. Agric. Food Chem..

[31] Liu H., Wang Z., Zhang D., Shen Q., Pan T., Hui T., Ma J. (2019). Characterization of key aroma compounds in Beijing roasted duck by gas chromatography–olfactometry–mass spectrometry, odor-activity values, and aroma-recombination experiments. J. Agric. Food Chem..

[32] Vera P., Canellas E., Nerín C. (2020). Compounds responsible for off-odors in several samples composed by polypropylene, polyethylene, paper and cardboard used as food packaging materials. Food Chem..

[33] Utama D.T., Kim Y.J., Jeong H.S., Kim J., Barido F.H., Lee S.K. (2020). Comparison of meat quality, fatty acid composition and aroma volatiles of dry-aged beef from Hanwoo cows slaughtered at 60 or 80 months old. Asian-Australas. J. Anim. Sci..

[34] Bruna J.M., Hierro E.M., de la Hoz L., Mottram D.S., Fernández M., Ordóñez J.A. (2003). Changes in selected biochemical and sensory parameters as affected by the superficial inoculation of *Penicillium camemberti* on dry fermented sausages. Int. J. Food Microbiol..

[35] Flores M., Corral S., Cano-García L., Salvador A., Belloch C. (2015). Yeast strains as potential aroma enhancers in dry fermented sausages. Int. J. Food Microbiol..

[36] Wen R., Sun F., Li X.-A., Chen Q., Kong B. (2021). The potential correlations between the fungal communities and volatile compounds of traditional dry sausages from Northeast China. Food Microbiol..

[37] Liang J., Xie J., Hou L., Zhao M., Zhao J., Cheng J., Wang S., Sun B.-G. (2016). Aroma constituents in Shanxi aged vinegar before and after aging. J. Agric. Food Chem..

[38] Frauendorfer F., Schieberle P. (2008). Changes in key aroma compounds of Criollo cocoa beans during roasting. J. Agric. Food Chem..

[39] Majcher M.A., Olszak-Ossowska D., Szudera-Kończal K., Jeleń H.H. (2020). Formation of key aroma compounds during preparation of pumpernickel bread. J. Agric. Food Chem..

[40] Niu Y., Zhang X., Xiao Z., Song S., Eric K., Jia C., Yu H., Zhu J. (2011). Characterization of odor-active compounds of various cherry wines by gas chromatography-mass spectrometry, gas chromatography-olfactometry and their correlation with sensory attributes. J. Chromatogr. B Anal. Technol. Biomed. Life Sci..

[41] Yang D.S., Shewfelt R.L., Lee K.-S., Kays S.J. (2008). Comparison of odor-active compounds from six distinctly different rice flavor types. J. Agric. Food Chem..

